# Diagnostic accuracy of the Clear Cell Likelihood Score and selected MRI parameters in the characterization of indeterminate renal masses – a single-institution study

**DOI:** 10.1007/s00261-024-04484-5

**Published:** 2024-07-09

**Authors:** Tomasz Blachura, Patrycja S. Matusik, Aleksander Kowal, Julia Radzikowska, Jarosław D. Jarczewski, Łukasz Skiba, Tadeusz J. Popiela, Robert Chrzan

**Affiliations:** 1 Department of Diagnostic Imaging, University Hospital, Kraków, 30-688 Poland; 2 Chair of Radiology, Jagiellonian University Medical College, Kraków, 30-688 Poland; 3 Department of Neurosurgery, Comprehensive Cancer Centre and Traumatology, Copernicus Memorial Hospital in Lodz, Lodz, Poland; 4 Student’s Scientific Group, Jagiellonian University Medical College, Kraków, 30-688 Poland

**Keywords:** ccLS score, RCC, Renal masses, Magnetic resonance imaging

## Abstract

**Purpose:**

We aimed to assess the diagnostic accuracy of the clear cell likelihood score (ccLS) and value of other selected magnetic resonance imaging (MRI) features in the characterization of indeterminate small renal masses (SRMs).

**Methods:**

Fifty patients with indeterminate SRMs discovered on MRI between 2012 and 2023 were included. The ccLS for the characterization of clear cell renal cell carcinoma (ccRCC) was calculated and compared to the final diagnosis (ccRCC vs. ‘all other’ masses).

**Results:**

The ccLS = 5 had a satisfactory accuracy of 64.0% and a very high specificity of 96.3%; however, its sensitivity of 26.1% was relatively low. Receiver operating curve (ROC) analysis revealed that from the selected MRI features, only T1 ratio and arterial to delayed enhancement (ADER) were good discriminators between ccRCC and other types of renal masses (area under curve, AUC = 0.707, *p* = 0.01; AUC = 0.673, *p* = 0.03; respectively). The cut-off points determined in ROC analysis using the Youden index were 0.73 (*p* = 0.01) for T1 ratio and 0.99 for ADER (*p* = 0.03). The logistic regression model demonstrated that ccLS = 5 and T1 ratio (OR = 15.5 [1.1-218.72], *p* = 0.04; OR = 0.002 [0.00-0.81], *p* = 0.04) were significant predictors of ccRCC.

**Conclusions:**

The ccLS algorithm offers an encouraging method for the standardization of imaging protocols to aid in the diagnosis and management of SRMs in daily clinical practice by enhancing detectability of ccRCC and reducing the number of unnecessary invasive procedures for benign or indolent lesions. However, its diagnostic performance needs multi-center large cohort studies to validate it before it can be incorporated as a diagnostic algorithm and will guide future iterations of clinical guidelines. The retrospective nature of our study and small patient population confined to a single clinical center may impact the generalizability of the results; thus, future studies are required to define whether employment of the T1 ratio or ADER parameter may strengthen the diagnostic accuracy of ccRCC diagnosis.

**Graphical Abstract:**

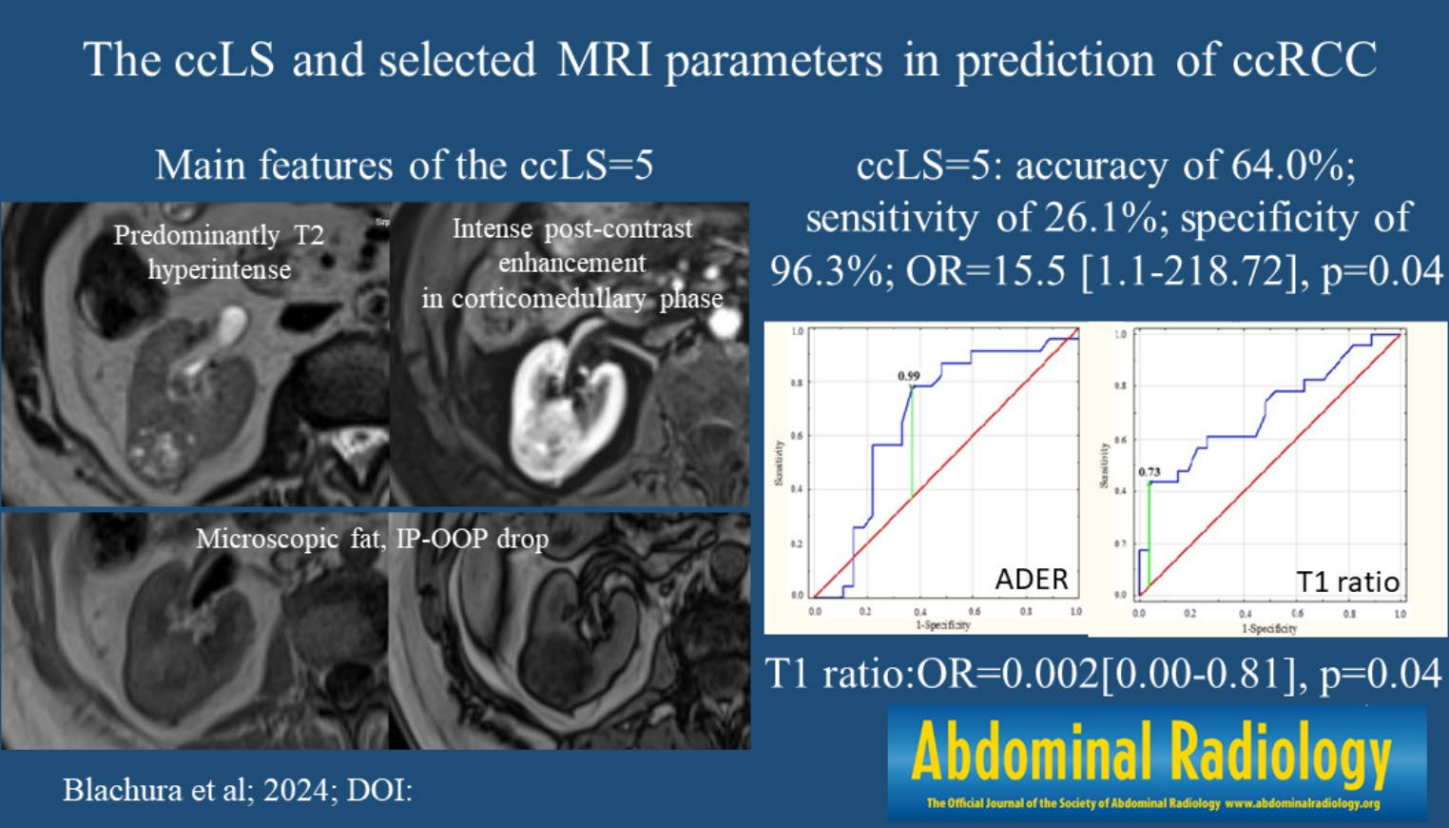

## Introduction

**Fig. 1:**
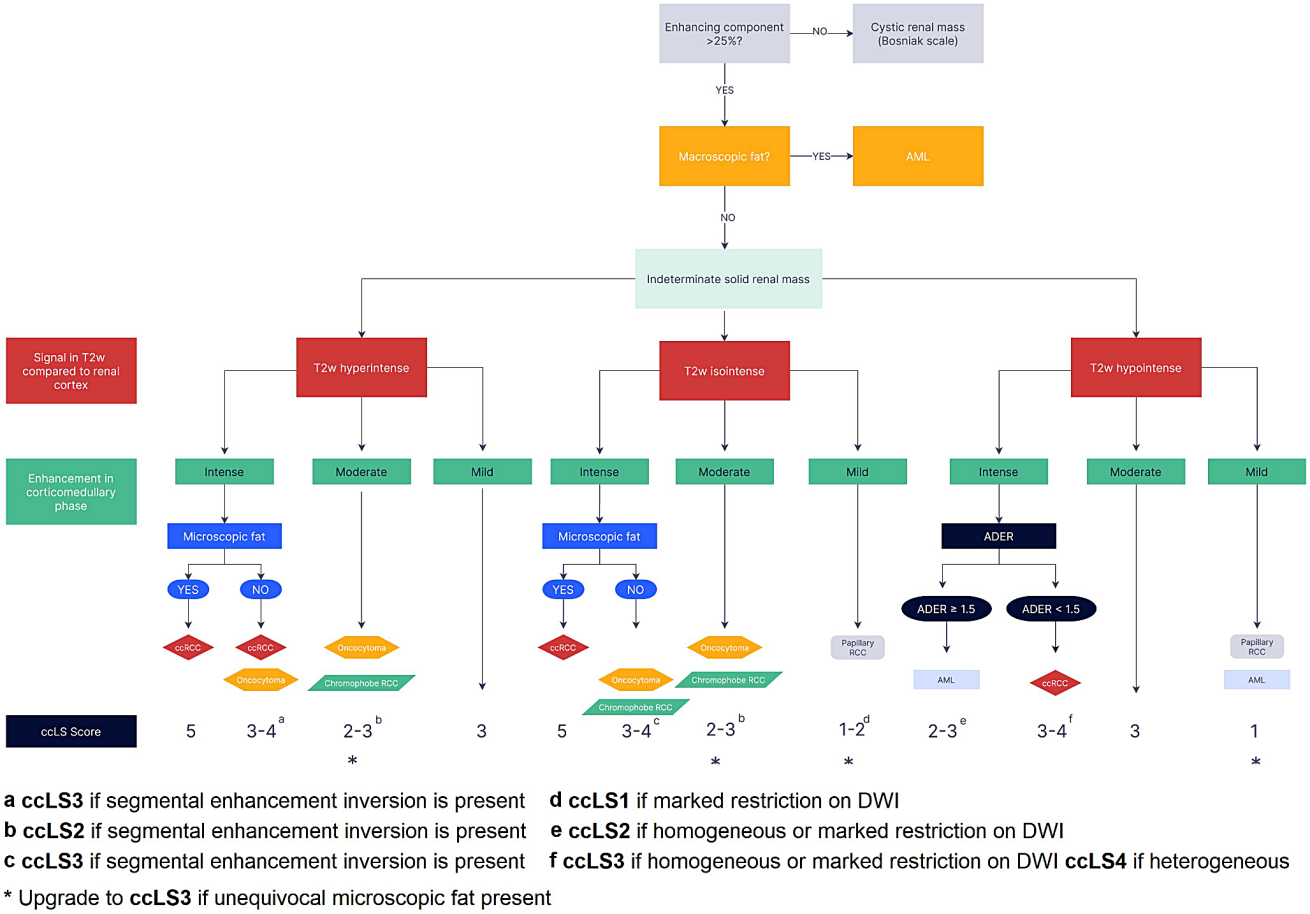
The ccLS algorithm. Adapted from Pedrosa I, Cadeddu JA. How We Do It: Managing the Indeterminate Renal Mass with the MRI Clear Cell Likelihood Score. Radiology. 2022 Feb;302(2):256–269; with permission. The ccLS assessment is based on three major imaging features (T2 signal intensity, level of post-contrast enhancement in corticomedullary phase compared to renal cortex, and the presence of microscopic fat) and several ancillary parameters

The clear cell likelihood score (ccLS) is a five-category Likert scale (from 1 point = very unlikely to 5 points = very likely) designed to enhance the prediction of histopathological characteristics of small renal masses (SRMs) in multiparametric magnetic resonance imaging (MRI) studies (Fig. 1) [1–3]. The ccLS assessment is based on three major imaging features (T2 signal intensity, level of post-contrast enhancement in corticomedullary phase compared to renal cortex, and the presence of microscopic fat) and several ancillary parameters [1, 4]. The algorithm is designed to provide measures for noninvasive detection or exclusion of the most aggressive form of renal carcinoma (RCC) – clear cell subtype (ccRCC) [5]. Precise differentiation between histopathologic subtypes is especially consequential for management planning since many postcontrast-enhancing renal tumors, including some papillary and chromophobe RCCs, exhibit an indolent behavior, with slow growth and only minimal risk of metastases [6, 7].

The ccLS is currently an interesting topic that has been studied and commented on extensively in the literature recently [1–5, 7–15]. Canvasser et al. revealed moderate to good inter-reader reliability of the ccLS algorithm (κ = 0.53) [16]. Schieda et al. recently published a large cohort, cross-sectional study validating diagnostic performance of the ccLS algorithm showing moderate sensitivity and specificity for diagnosis of ccRCC with a threshold of ccLS ≥ 4 and high negative predictive value of ccLS ≤ 2 to exclude ccRCC [17]. Tian et al. noted that state-of-the-art studies demonstrated moderate sensitivity and specificity for the ccLS algorithm; nevertheless, the algorithm was effective in the context of clinical application [18]. Available data in this area predominantly is from retrospective, single-center studies which were conducted on a limited population (ranged from 57 to 241 patients). The existing literature has also highlighted certain limitations of the algorithm within specific diagnostic domains, e.g. the ccLS does not consider the papillary and chromophobe subtypes of RCC [18]. This suggests that some improvements should be considered in the future version of ccLS, e.g. adding parameters, which may indicate significant post-contrast enhancement, such as low T1 ratio or high arterial to delayed enhancement (ADER) [19, 20].

Therefore, we attempted to further validate the ccLS algorithm in a new patient cohort and investigated the value of other selected MRI features, especially related with post-contrast enhancement in the prediction of ccRCC occurrence.

## Methodology

### Studied population

In this retrospective, observational, single-center study, we included *N* = 50 patients with indeterminate SRMs discovered on MRI between 2012 and 2023. Final diagnoses were made using histopathologic results as standard reference (*n* = 38). In 12 patients, the final diagnosis was established based on clinical and imaging features (diagnosis of lipid-poor angiomyolipoma or non-ccRCC masses without exact subtype information) if a regression or complete lack of progression was observed during follow-up (minimum 36 months, mean 45.58 ± 9 months) [6, 21–24]. Exclusion criteria were as follows: diffuse infiltrative renal disease (i.e. lymphoma), masses containing macroscopic fat, predominantly cystic mass, MRI artifacts that could compromise renal lesion segmentation, acute intralesional complications (e.g. hemorrhage), incomplete clinical data, and inability to make a definitive diagnosis due to ambiguous radiological image and lack of histopathology. The study was approved by the Ethics Committee.

### Magnetic resonance imaging studies

Magnetic resonance imaging studies were performed in the Department of Diagnostic Imaging in the University Hospital of Cracow, both on 1.5T and 3.0T scanners, using protocols meeting the requirements of ccLS algorithm application and according to the Society of Abdominal Radiology Disease expert consensus. All MRIs included conventional sequences: axial and coronal 2D T2w single shot acquisitions, axial 2D T1w gradient echo in and out of phase for chemical shift imaging, pre- and (dynamic) post-contrast 3D T1w SPGR with fat saturation including delayed scans and diffusion-weighted imaging. All MRI examinations were retrospectively reviewed by two radiologists (T.B. and J.D.J) with at least 3 years experience and were blinded to the final diagnosis. The inter-reader reliability of the ccLS algorithm in our study was good (κ = 0.63). We used the ccLS system to provide a standardized framework for categorizing small renal masses [9], as well as conducted separate evaluation of selected imaging parameters, both those present in the ccLS algorithm and potentially useful new parameters. The following MRI parameters were used for our analysis: T1 and T2 signal intensity (SI) ratio (measured as the ratio of tumor SI to renal cortex SI), intensity of multiphasic contrast enhancement, chemical shift imaging for the presence of microscopic fat, diffusion restriction, ADER (obtained as the ratio of difference between signal intensity in the corticomedullary phase and pre-contrast images to the difference in signal intensity in delayed phase and pre-contrast images), and segmental enhancement inversion (defined as the presence of relative inversion of post-contrast enhancement degree in corticomedullary and later phases).

### Statistical analysis

Scoring each renal mass for ccRCC diagnosis was compared to the reference standard, which was determined by histology or imaging (ccRCC vs. ‘all other’ masses). Receiver operating characteristic (ROC) analysis was performed to calculate the area under the curve (AUC) and was used to determine which of the selected MRI features are discriminators between ccRCC and other types of SRMs. A logistic regression was performed in order to determine which of the MRI parameters are significant predictors of ccRCC. The Student’s T-test or Mann-Whitney U test were used to assess differences in continuous variables between ccRCC and non-ccRCC, when appropriate. Continuous variables were expressed as means ± standard deviations or medians and interquartile ranges (IQR). The Pearson χ^2^ test was used for investigation of associations between categorical variables. Categorical variables were given as numbers and percentages. A p-value of 0.05 or less was considered statistically significant. Specificity, sensitivity, positive predictive value, negative predictive value, accuracy, and likelihood ratio were calculated for each ccLS. Statistical analyses were performed using IBM SPSS Statistics (version 24, IBM Corp., Armonk, NY, USA). Confidence intervals (CI) were calculated using MEDCALC (free statistical calculators).

## Results

### Patient characteristics

A total of 50 patients with indeterminate renal masses (27 females, 23 males; median age 61.5 [IQR: 52.3–68.3] years) were retrospectively enrolled in the study. In 70.0% of patients, hypertension was observed, and 44.0% had a documented history of cigarette smoking. Symptomatic presentations were noted in 20.0% of the cases: flank pain (14.0%), microhematuria (8.0%), macrohematuria (6.0%), polycythemia (4.0%); no reports of palpable flank mass or paraneoplastic syndromes. A positive family history of cancer was observed in 20% of the patients. In the ccRCC group, there were significantly more cigarette smokers than in the non-ccRCC group (14 [60.9%] vs. 8 [29.6%], *p* = 0.03). Apart from this, the ccRCC and non-ccRCC groups did not differ in main risk factors related with ccRCC development (Table 1).

**Table 1:** Prevalence of renal carcinoma risk factors in patients with confirmed ccRCC and group with other etiologies of renal masses

Risk factors	ccRCC (*n* = 23)	non-ccRCC (*n* = 27)	*p*-value
Male sex	10 (43.5%)	13 (48.1%)	0.74
Symptoms	4 (17.4%)	6 (22.2%)	0.67
Smoking	14 (60.9%)	8 (29.6%)	0.03
Hypertension	18 (78.3%)	17 (63.0%)	0.24
Family history of neoplasm	11 (47.8%)	9 (33.3%)	0.30

*Abbreviations* ccRCC – clear cell renal cell carcinoma; non-ccRCC – other than clear cell renal cell carcinoma

### MRI features in ccRCC and non-ccRCC groups

When comparing MRI features, only median values of the T1 ratio and ADER differ significantly between ccRCC and non-ccRCC groups. Median (IQR) of the T1 ratio was significantly lower in the ccRCC group than in the non-ccRCC group (0.8 [0.7–0.9] vs. 0.9 [0.9-1.0], *p* = 0.01). On the other hand, median (IQR) of the ADER was significantly greater in the ccRCC group when compared to other renal mass etiologies (1.1 [1.0-1.4] vs. 0.9 [0.6–1.1], *p* = 0.04). All evaluated MRI features in ccRCC and non-ccRCC groups are presented in Table 2.

**Table 2:** Differences in MRI features in ccRCC group and patients with other etiology of renal masses

MRI features	ccRCC (*n* = 23)	non-ccRCC (*n* = 27)	*p*-value
Diffusion restriction	12 (52.2%)	17 (63.0%)	0.44
Segmental enhancement inversion	0 (0.0%)	2 (7.4%)	0.18
Arterial to delayed enhancement ratio > 1.5	1 (4.3%)	3 (11.1%)	0.38
Arterial to delayed enhancement ratio	1.1 [1.0-1.4]	0.9 [0.6–1.1]	**0.04**
Microscopic fat	5 (21.7%)	4 (14.8%)	0.53
T2 hyperintensity	12 (52.2%)	12 (44.4%)	0.59
T2 SI ratio	1.1 [0.9–1.5]	1.1 [0.8–1.6]	0.65
T1 SI ratio	0.8 [0.7–0.9]	0.9 [0.9-1.0]	**0.01**
Cortical hiperenhancement	17 (73.9%)	14 (51.9%)	0.11
Largest axial measurement (mm)	22.0 [19.0–29.0]	17.0 [13–26]	0.07
Largest cranio-caudal measurement (mm)	26.0 [17.0–28.0]	16.0 [10–24]	0.08
% of solid mass	80.0 [30–100]	100.0 [40.0-100.0]	0.15

*Abbreviations* ccRCC – clear cell renal cell carcinoma; non-ccRCC – other than clear cell renal cell carcinoma; SI – signal intensity

### Diagnostic accuracy of ccLS scores in the prediction of ccRCC and other masses

Patients with ccRCC differ significantly from patients with non-ccRCC only regarding ccLS = 1/2 and ccLS = 5 (1 [4.3%] vs. 8 [29.6%], *p* = 0.02; 6 [26.1%] vs. 1 [3.7%], *p* = 0.02; respectively, Table 3). Sensitivity, specificity, and other parameters for particular ccLS scores in the prediction of ccRCC are described in Table 4. The best performance parameter was ccLS = 4/5 with an accuracy of 60.0%, sensitivity of 59.3%, and specificity of 60.9%. The ccLS = 5 had a satisfactory accuracy of 64.0% and very high specificity of 96.3%; however, its sensitivity of 26.1% was too low.

**Table 3:** Differences in ccLS scores between patients with ccRCC and other renal masses

ccLS score	ccRCC (*n* = 23)	non-ccRCC (*n* = 27)	*p*-value
ccLS = 1	1 (4.3%)	7 (26.0%)	0.06
ccLS = 2	0 (0.0%)	1 (3.7%)	0.35
ccLS = 1/2	1 (4.3%)	8 (29.6%)	0.02
ccLS = 3	8 (34.8%)	8 (29.6%)	0.70
ccLS = 3/4	16 (69.6%)	18 (66.7%)	0.15
ccLS = 4	8 (34.8%)	10 (37.0%)	0.87
ccLS = 4/5	14 (60.9%)	11 (40.7%)	0.16
ccLS = 5	6 (26.1%)	1 (3.7%)	0.02

*Abbreviations* ccLS – clear cell likelihood score; ccRCC – clear cell renal cell carcinoma; non-ccRCC – other than clear cell renal cell carcinoma

**Table 4:** Diagnostic performance for particular ccLS scores

Score	Accuracy	Sensitivity	Specificity	PLR	NLR	PPV	NPV
ccLS = 1	42.0 (29.2–56.8)%	4.3 (0.1–22.0)%	74.1 (53.7–88.9)%	0.17 (0.0-1.3)	1.29 (1.0-1.6)	12.5 (1.9–51.9)%	47.6 (41.7–53.6)%
ccLS = 2	52.0 (37.4–66.3)%	0.0%*	96.3 (81.0-99.9)%	0.0*	1.04 (1.0-1.1)	0.0 (0.0-14.8)%	53.1 (51.2–54.9)%
ccLS = 1/2	40.0 (26.4–54.8)%	4.3 (0.1–22.0)%	70.4 (49.8–86.3)%	0.15 (0.0-1.1)	1.36 (1.1–1.8)	11.1 (1.7–48.1)%	46.3 (40.0-52.8)%
ccSL = 3	54.0 (39.3–68.2)%	34.8 (16.4–57.3)%	70.4 (49.8–86.3)%	1.17 (0.5–2.6)	0.93 (0.6–1.4)	50.0 (30.9–69.1)%	55.9 (46.3–65.1)%
ccLS = 3/4	50.0 (35.5–64.5)%	69.6 (47.1–86.8)%	33.3 (16.5–54.0)%	1.04 (0.7–1.5)	0.91 (0.4–2.1)	47.1 (37.8–56.5)%	56.3 (36.2–74.4)%
ccLS = 4	50.0 (35.5–64.5)%	34.8 (16.4–57.3)%	63.0 (42.4–80.6)%	0.9 (0.5-2.0)	1.04 (0.7–1.6)	44.4 (27.5–62.8)%	53.1 (42.8–63.2)%
ccLS = 5	64.0 (49.2–77.1)%	26.1 (10.2–48.4)%	96.3 (81.0-99.9)%	7.04 (0.9–54.3)	0.77 (0.6-1.0)	85.7 (43.8–97.9)%	60.5 (54.3–66.3)%
ccLS = 4/5	60.0 (45.2–73.6)%	60.9 (38.5–80.3)%	59.3 (38.8–77.6)%	1.49 (0.9–2.6)	0.66 (0.4–1.2)	56.0 (42.1–69.0)%	64.0 (49.4–76.4)%

*Abbreviations* ccLS – clear cell likelihood score; PLR – positive likelihood ratio; NLR – negative likelihood ratio; PPV – positive predictive value; NPV – negative predictive value

*CI not available

### Selected MRI features in the prediction of ccRCC

**Fig. 2:**
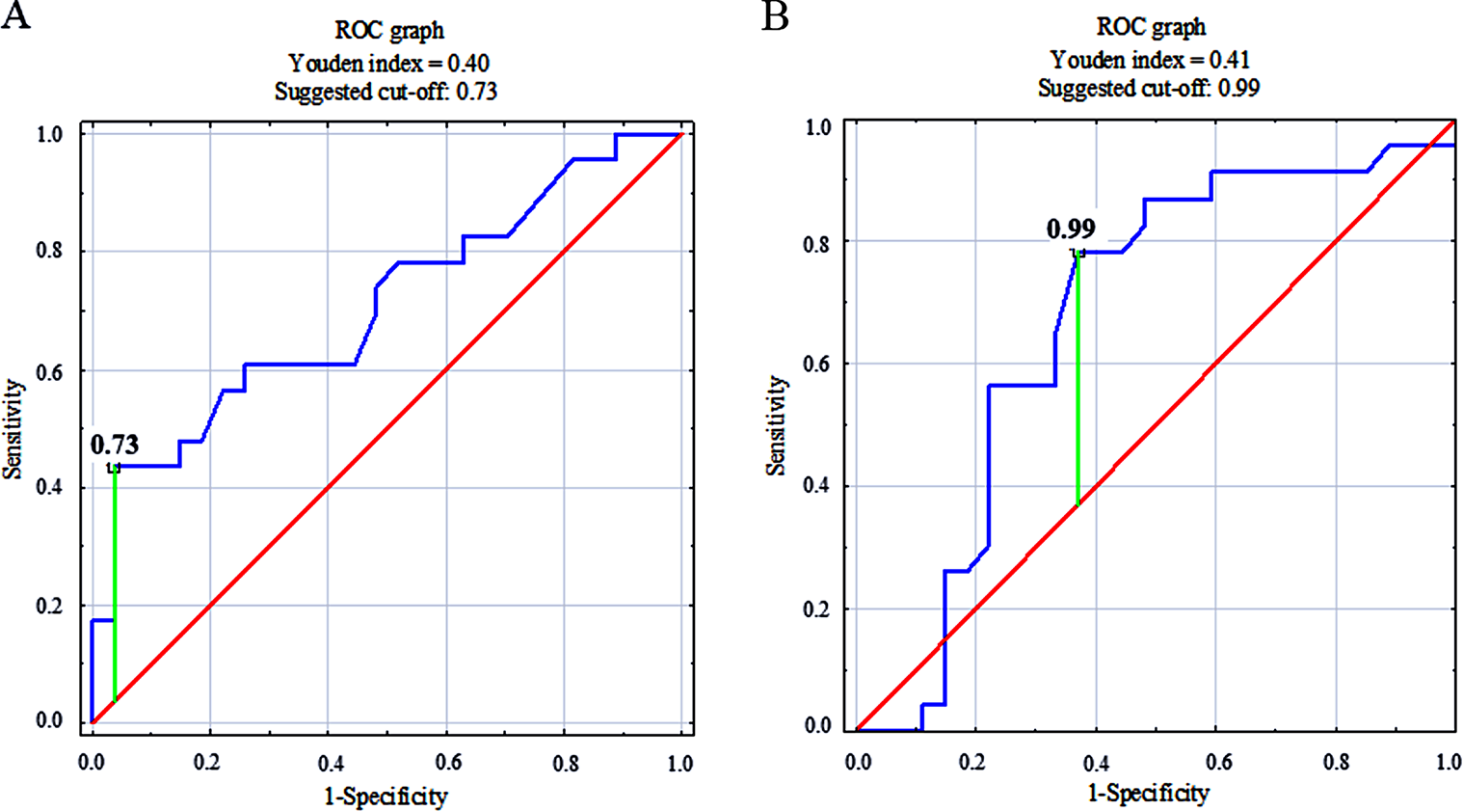
ROC analysis of the T1 ratio (**A**) and the ADER (**B**). T1 ratio - the ratio of tumor SI to renal cortex SI; ADER - arterial to delayed ratio, obtained as the ratio of difference between signal intensity in the corticomedullary phase and pre-contrast images to the difference in signal intensity in delayed phase and pre-contrast images

Analysis of ROC revealed that from the selected MRI features only the T1 ratio and ADER were discriminators between ccRCC and other types of renal masses (Fig. 2; Table 5). The cut-off points determined in our ROC analysis using the Youden index were 0.73 (*p* = 0.01) for the T1 ratio, and 0.99 for ADER (*p* = 0.03).

**Table 5:** Area under the curve analyses according to selected MRI parameters

MRI features	AUC	*p*-value
T1 SI ratio	0.707	**0.01**
ADER	0.673	**0.03**
T2 SI ratio	0.538	0.65
Largest axial measurement (mm)	0.650	0.49
Largest cranio-caudal measurement (mm)	0.643	0.49
% of solid mass	0.386	0.17

Data are presented as the area under the curve (p-value). Abbreviations: AUC – area under the curve; SI – signal intensity; ADER - arterial to delayed enhancement ratio

The logistic regression model demonstrated that ccLS = 5 (OR = 15.5, CI = 1.1-218.72, *p* = 0.04) was a significant predictor of ccRCC (Table 6). Interestingly, increasing values of the T1 ratio (OR = 0.002, CI:0.00-0.81, *p* = 0.04) significantly correspond to decreasing odds of the ccRCC occurrence.

**Table 6:** Multivariate logistic regression model predicting an occurrence of ccRCC

MRI features	OR (95% CI)	*p*-value
T1 SI ratio	0.002 (0.00-0.81)	**0.04**
ADER	1.32 (0.32–5.37)	0.70
T2 SI ratio	0.47 (0.08–2.67)	0.39
Largest axial measurement (mm)	0.86 (0.70–1.06)	0.15
Largest cranio-caudal measurement (mm)	1.21 (0.99–1.49)	0.06
% of solid mass	1.00 (0.97–1.02)	0.91
ccLS = 4	1.75 (0.33–9.39)	0.52
ccLS = 5	15.5 (1.1-218.72)	**0.04**

*Abbreviations* ADER - arterial to delayed enhancement ratio; ccLS – clear cell likelihood score; CI - confidence interval; OR – odds ratio; SI – signal intensity

## Discussion

The main goal of our study was validation of the ccLS algorithm. We demonstrated that only ccLS = 5 was a significant predictor of ccRCC occurrence, with a specificity of 96.3% and sensitivity of 26.1%. Unfortunately, in our study, 73.9% of ccRCC cases were classified outside the ccLS = 5 group (reflecting low sensitivity of ccLS = 5), most of them in ccLS = 3 and ccLS = 4 groups, where the ccLS algorithm has lower diagnostic confidence. Previous studies have shown that the ccLS had a moderate to high accuracy for differentiating ccRCC from other small renal masses [13, 16, 18, 25]. A recently published meta-analysis has demonstrated that the sensitivity and specificity of ccLS ≥ 4 for risk stratification of ccRCC were 0.75–0.89 and 0.58–0.82 for individual studies, respectively [18]. These data regarding the specificity are consistent with our results. The difference in observed sensitivity may result from the adopted cut-off point of ccLS (ccLS ≥ 4 in meta-analysis and ccLS = 5 in our study). For ccLS = 4/5 we observed an accuracy of 60.0%, sensitivity of 59.3%, and specificity of 60.9%. However, this cut-off point did not reach the level of statistical significance, probably due to the small sample size included in our study.

Historically, the assessment of SRMs was based mainly on computer tomography imaging and was binary, confined to confirming post-contrast enhancement and excluding the presence of macroscopic fat within the lesion, and with limited differentiation of the histopathological nature of the tumor [26]. Various methods have been proposed for augmenting this fundamental assessment by differentiating tumor types [27], including radiomics-based models for differentiating ccRCC from other SRMs [28, 29]. Compared to computer tomography, MRI provides superior tissue resolution and intravenous contrast response, as well as the simultaneous capability of detecting microscopic fat, potentially rendering better results in prediction of the histopathologic type of lesion. Apart from the ccLS, various MRI algorithms for ccRCC differentiation were created; however, to the best of our knowledge, validation of these algorithms has not been performed [30, 31]. In light of our findings, further improvements and increased utilization of the ccLS algorithm may enhance the role of active surveillance as initial management in selected SRMs as opposed to invasive management; thus, reducing the burden of surgical complications and negative effects of nephron loss.

Interestingly, our study showed that values of the T1 ratio were significantly lower in the ccRCC group than in the non-ccRCC group (0.8 [0.7–0.9] vs. 0.9 [0.9-1.0]), and that a low T1 SI ratio (< 0.73) was a good discriminator between ccRCC and other types of renal masses in ROC analysis. Moreover, logistic regression analysis revealed that increasing values of the T1 ratio (OR = 0.002, CI:0.00-0.81, *p* = 0.04) correspond to decreasing odds of ccRCC occurrence. However, confirmation of this data requires further research on a larger group of patients. Currently, T1-weighted acquisitions are used in the ccLS algorithm only in chemical shift imaging and multiphasic contrast-enhanced imaging [9]. In light of our results, employment of direct values of the T1 SI ratio may optimize diagnostic confidence of this algorithm; however, further studies in this area are needed. Specifically, papillary RCC tumors require testing, since it has been previously demonstrated that mean tumor T1 SI ratios of papillary RCCs (reader 1, 0.86 ± 0.23; reader 2, 0.82 ± 0.30) were not significantly different from those of ccRCCs (reader 1, 0.81 ± 0.15; reader 2, 0.84 ± 0.27) [28]. Papillary RCC, the second most common RCC variant, usually presents with both low T1 SI and T2 SI (with a tumor T2 SI ratio of ≤ 0.66 having a specificity of 100% and sensitivity of 54% for papillary RCC) [32, 33]. Therefore, in cases of papillary RCC, a low T1 SI may be an especially useful parameter in the assessment of ccRCC risk in lesions with a moderate to high T2 SI.

Our analysis also confirmed the importance of the ADER parameter, showing a statistically significant high specificity of intermediate values of ADER in the prediction of ccRCC, with a mean ADER = 1.1 [1.0-1.4] for ccRCC and a mean ADER = 0.9 [0.6–1.1] for non-ccRCC. During ccLS assessment, ADER > 1.5 is mainly used for differentiation of ccRCC from fat-poor angiomyolipoma, which is a lesion with a high “washout” [34]. Our results correspond to observations that ccRCC tends to show moderate “washout” and demonstrate that ccRCC may be differentiated from other forms of renal masses with a lower cut-off point of ADER = 0.99. However, this cut-off point should be validated in larger populations.

**Fig. 3:**
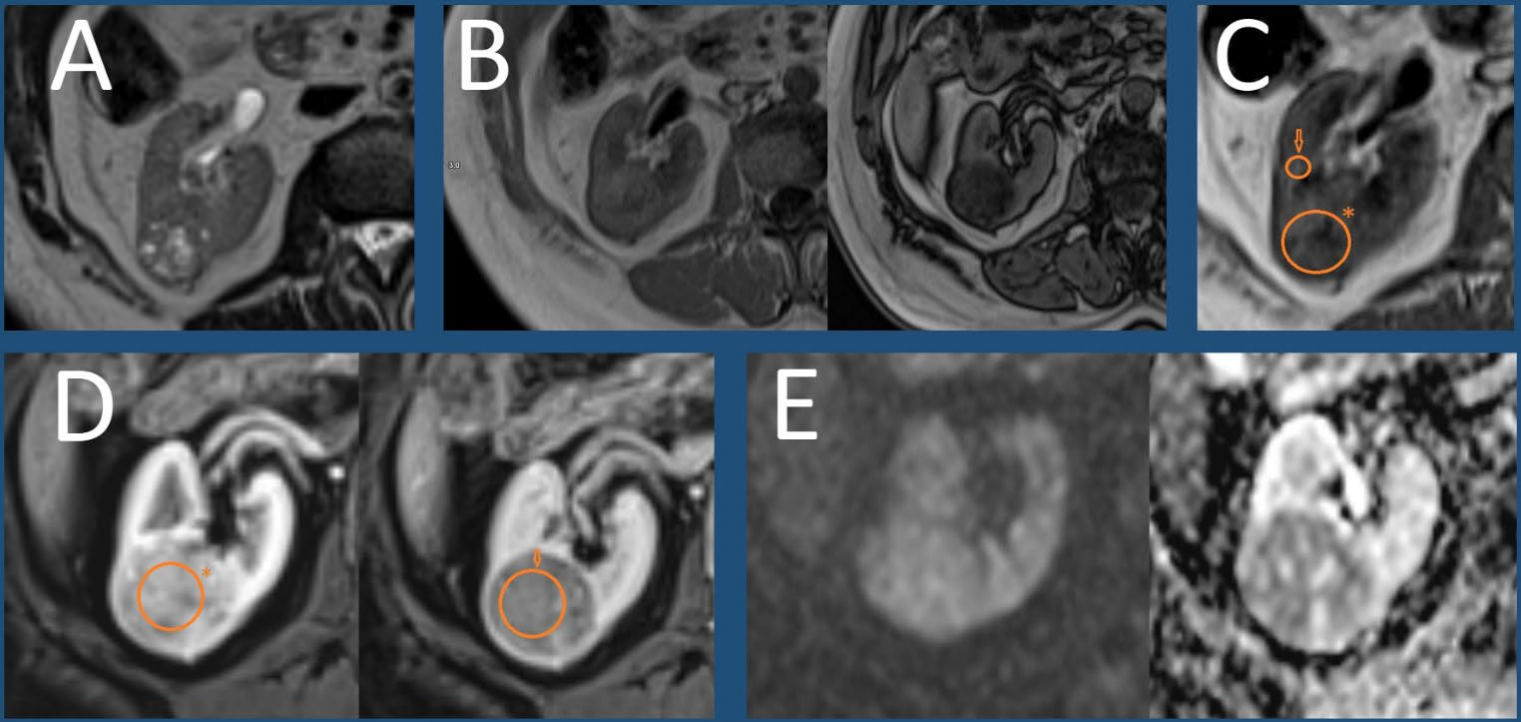
A clinical case showing the implementation of T1 and ADER parameters and utilization of the ccLS score. Axial images of the right kidney show a posteriorly localized, well-defined, oval mass in the right kidney of a 78-year-old female patient. The lesion was subsequently resected and diagnosed as clear cell renal cell carcinoma using histopathological verification. (**A**) T2-weighted acquisition shows a heterogeneous, mostly T2-hyperintense mass with areas of isointense signal (note that a T2-isointense lesion with similar characteristics would also be classified as ccLS = 5) (**B**) Two T1-weighted Dixon acquisitions at the same level show a significant decrease in signal intensity on opposed-phase image (right) compared with in-phase images (left) (**C**) T1-weighted image showing a heterogeneous, slightly hypointense lesion (ROI with *) compared to normal renal cortex (ROI with arrow) with T1 SI ratio = 0.87 (**D**) Two fat-saturated T1-weighted postcontrast images at the same level in corticomedullary (left) and delayed (right) phases show significant early enhancement (ROI with *) and signal drop in delayed phase (ROI with arrow) with ADER = 1.2 (**E**) Two DWI (left) and ADC map (right) images at the same level show diffusion restriction in the tumor compared to renal cortex (note that restriction at DWI is considered only an ancillary feature in the ccLS algorithm and in many cases it is more suggestive of indolent lesions rather than ccRCC. In this case, the tumor would be considered ccLS = 5, regardless of its DWI signal)

Our observations regarding T1 and ADER parameters will undoubtedly require validation in studies with a larger cohort and under multi-center conditions. If subsequent research confirms their effectiveness, the above parameters may aid in clinical decision-making regarding surgical procedures or biopsy verification, or they might even contribute to the construction of a new, modified scale. Figure 3 demonstrates a clinical case showing the implementation of T1 and ADER parameters and utilization of the ccLS score.

Our study has several limitations. First, it is retrospective, which may lead to a high risk of bias in terms of the patient selection domain. Additionally, it is only a single-institution series and not all patients who had MRI underwent surgery or biopsy. Currently, there is a lack of prospective data comparing available surveillance strategies. This results in essential variability in the approach and in varied frequency and duration of follow-up imaging. However, there are studies regarding active surveillance for patients with SRMs with a follow-up time of 3 years [6, 21–24]. Therefore, the minimum follow-up time of patients without surgery or biopsy in our study was stated as 36 months [6, 21–24]. Diagnostic accuracy of MRI studies could vary based on the radiologist’s experience (given the somewhat subjective nature of assigning the ccLS score). Without randomization of management (surveillance, biopsy, and surgery), there is an inherent selection bias in which patients had histopathology available. Lastly, our study has a small cohort of only 50 renal masses, of which 23 were ccRCC.

## Conclusions

The ccLS algorithm has moderate sensitivity and specificity for the evaluation of ccRCC. These findings suggest that the ccLS algorithm should be still validated and tested before it can be incorporated as a diagnostic algorithm, and will guide future iterations of clinical guidelines. Employment of direct values of the T1 ratio parameter may strengthen the diagnostic accuracy of ccRCC diagnosis. However, further studies in this area performed on large and multi-center cohorts are required. The continued development and widespread acceptance of the ccLS algorithm may in the near future reduce the reliance on biopsies in the diagnosis of renal tumors, enhance the early detection of the clear cell variant of RCC, and decrease the burden of avoidable surgical procedures in the management of SRMs by replacing them with an active surveillance strategy.

## References

[1] Pedrosa, I. and J.A. Cadeddu, *How We Do It: Managing the Indeterminate Renal Mass with the MRI Clear Cell Likelihood Score*. Radiology, 2022. 302(2): p. 256–269. 34904873 10.1148/radiol.210034 PMC8805575

[2] Tse, J.R., *Editorial Comment: Clear Cell Likelihood Score-Another Step Toward Noninvasive Risk Stratification*. AJR Am J Roentgenol, 2022. 219(5): p. 803. 35703414 10.2214/AJR.22.28087

[3] Mileto, A. and T.A. Potretzke, *Standardized Evaluation of Small Renal Masses Using the MRI Clear Cell Likelihood Score*. Radiology, 2022. 303(3): p. 600–602. 35289666 10.1148/radiol.220054

[4] Hao, Y., S. Gao, X. Zhang, et al., *[Comparison of diagnostic performance of Clear Cell Likelihood Score v1.0 and v2.0 for clear renal cell carcinoma]* Nan Fang Yi Ke Da Xue Xue Bao, 2023. 43(5): p. 800–806. 10.12122/j.issn.1673-4254.2023.05.16 PMC10267243 37313822

[5] Hao, Y.W., Y. Zhang, H.P. Guo, et al., *Differentiation between renal epithelioid angiomyolipoma and clear cell renal cell carcinoma using clear cell likelihood score*. Abdom Radiol (NY), 2023. 48(12): p. 3714–3727. 37747536 10.1007/s00261-023-04034-5

[6] Smaldone, M.C., A. Kutikov, B.L. Egleston, et al., *Small renal masses progressing to metastases under active surveillance: a systematic review and pooled analysis*. Cancer, 2012. 118(4): p. 997–1006. 21766302 10.1002/cncr.26369 PMC4329724

[7] Vazquez, L.C., Y. Xi, R.G. Rasmussen, et al., *Characterization of Demographical Histologic Diversity in Small Renal Masses With the Clear Cell Likelihood Score*. J Comput Assist Tomogr, 2024. 10.1097/RCT.0000000000001567 38213063

[8] Chen, K.Y., M.J. Lange, J.X. Qiu, et al., *Cost-Effectiveness Analysis of the Clear Cell Likelihood Score against Renal Mass Biopsy for Evaluating Small Renal Masses* Urology, 2024. 10.1016/j.urology.2024.04.007 PMC11193637 38648945

[9] Shetty, A.S., T.J. Fraum, D.H. Ballard, et al., *Renal Mass Imaging with MRI Clear Cell Likelihood Score: A User’s Guide*. Radiographics, 2023. 43(7): p. e220209. 37319026 10.1148/rg.220209

[10] Pedrosa, I., *Invited Commentary: MRI Clear Cell Likelihood Score for Indeterminate Solid Renal Masses: Is There a Path for Broad Clinical Adoption?* Radiographics, 2023. 43(7): p. e230042. 37319027 10.1148/rg.230042 PMC10323227

[11] Shetty, A.S., *Editorial Comment: Toward a CT Equivalent of the MRI Clear Cell Likelihood Score*. AJR Am J Roentgenol, 2022. 219(5): p. 824. 35766536 10.2214/AJR.22.28118

[12] Rasmussen, R.G., Y. Xi, R.C. Sibley, 3rd, et al., *Association of Clear Cell Likelihood Score on MRI and Growth Kinetics of Small Solid Renal Masses on Active Surveillance*. AJR Am J Roentgenol, 2022. 218(1): p. 101–110. 34286596 10.2214/AJR.21.25979 PMC8725913

[13] Dunn, M., V. Linehan, S.E. Clarke, et al., *Diagnostic Performance and Interreader Agreement of the MRI Clear Cell Likelihood Score for Characterization of cT1a and cT1b Solid Renal Masses: An External Validation Study*. AJR Am J Roentgenol, 2022. 219(5): p. 793–803. 35642765 10.2214/AJR.22.27378

[14] Cui, M.Q., B. He, W. Xu, et al., *[Value of clear cell likelihood score in differentiation between renal oncocytoma and clear cell renal cell carcinoma]*. Zhonghua Yi Xue Za Zhi, 2022. 102(47): p. 3779–3785. 36517429 10.3760/cma.j.cn112137-20221020-02193

[15] Diaz de Leon, A., M.S. Davenport, S.G. Silverman, et al., *Role of Virtual Biopsy in the Management of Renal Masses*. AJR Am J Roentgenol, 2019. 212(6): p. 1234–1243. 30995092 10.2214/AJR.19.21172 PMC6800762

[16] Canvasser, N.E., F.U. Kay, Y. Xi, et al., *Diagnostic Accuracy of Multiparametric Magnetic Resonance Imaging to Identify Clear Cell Renal Cell Carcinoma in cT1a Renal Masses*. J Urol, 2017. 198(4): p. 780–786. 28457802 10.1016/j.juro.2017.04.089 PMC5972826

[17] Schieda, N., M.S. Davenport, S.G. Silverman, et al., *Multicenter Evaluation of Multiparametric MRI Clear Cell Likelihood Scores in Solid Indeterminate Small Renal Masses*. Radiology, 2023. 306(3): p. e239001. 36803006 10.1148/radiol.239001

[18] Tian, J., F. Teng, H. Xu, et al., *Systematic review and meta-analysis of multiparametric MRI clear cell likelihood scores for classification of small renal masses*. Front Oncol, 2022. 12: p. 1004502. 36387185 10.3389/fonc.2022.1004502 PMC9641245

[19] Le, J., M. Flusberg, A.M. Rozenblit, and V. Chernyak, *T1-hyperintense renal lesions: can high signal predict lack of enhancement?* Abdom Imaging, 2015. 40(8): p. 3175–3181. 26423276 10.1007/s00261-015-0539-0

[20] Moldovanu, C.G., B. Petresc, A. Lebovici, et al., *Differentiation of Clear Cell Renal Cell Carcinoma from other Renal Cell Carcinoma Subtypes and Benign Oncocytoma Using Quantitative MDCT Enhancement Parameters*. Medicina (Kaunas), 2020. 56(11). 10.3390/medicina56110569 PMC7692100 33126571

[21] Campbell, S.C., P.E. Clark, S.S. Chang, et al., *Renal Mass and Localized Renal Cancer: Evaluation, Management, and Follow-Up: AUA Guideline: Part I*. J Urol, 2021. 206(2): p. 199–208. 34115547 10.1097/JU.0000000000001911

[22] Campbell, S.C., R.G. Uzzo, J.A. Karam, et al., *Renal Mass and Localized Renal Cancer: Evaluation, Management, and Follow-up: AUA Guideline: Part II*. J Urol, 2021. 206(2): p. 209–218. 34115531 10.1097/JU.0000000000001912

[23] Kunkle, D.A., B.L. Egleston, and R.G. Uzzo, *Excise, ablate or observe: the small renal mass dilemma–a meta-analysis and review*. J Urol, 2008. 179(4): p. 1227–1233; discussion 1233 – 1224. 18280512 10.1016/j.juro.2007.11.047

[24] Chawla, S.N., P.L. Crispen, A.L. Hanlon, et al., *The natural history of observed enhancing renal masses: meta-analysis and review of the world literature*. J Urol, 2006. 175(2): p. 425–431. 16406965 10.1016/S0022-5347(05)00148-5

[25] Schieda, N., M.S. Davenport, S.G. Silverman, et al., *Multicenter Evaluation of Multiparametric MRI Clear Cell Likelihood Scores in Solid Indeterminate Small Renal Masses*. Radiology, 2022. 303(3): p. 590–599. 35289659 10.1148/radiol.211680 PMC9794383

[26] Bazzocchi, M.V., C. Zilioli, V.I. Gallone, et al., *The Role of CT Imaging in Characterization of Small Renal Masses*. Diagnostics (Basel), 2023. 13(3). 10.3390/diagnostics13030334 PMC9914376 36766439

[27] Lee-Felker, S.A., E.R. Felker, N. Tan, et al., *Qualitative and quantitative MDCT features for differentiating clear cell renal cell carcinoma from other solid renal cortical masses*. AJR Am J Roentgenol, 2014. 203(5): p. W516-524. 25341166 10.2214/AJR.14.12460

[28] Kim, T.M., H. Ahn, H.J. Lee, et al., *Differentiating renal epithelioid angiomyolipoma from clear cell carcinoma: using a radiomics model combined with CT imaging characteristics*. Abdom Radiol (NY), 2022. 47(8): p. 2867–2880. 35697856 10.1007/s00261-022-03571-9

[29] Qu, J., Q. Zhang, X. Song, et al., *CT differentiation of the oncocytoma and renal cell carcinoma based on peripheral tumor parenchyma and central hypodense area characterisation*. BMC Med Imaging, 2023. 23(1): p. 16. 36707788 10.1186/s12880-023-00972-0 PMC9881251

[30] Cornelis, F. and N. Grenier, *Multiparametric Magnetic Resonance Imaging of Solid Renal Tumors: A Practical Algorithm*. Semin Ultrasound CT MR, 2017. 38(1): p. 47–58. 28237280 10.1053/j.sult.2016.08.009

[31] Sun, M.R., L. Ngo, E.M. Genega, et al., *Renal cell carcinoma: dynamic contrast-enhanced MR imaging for differentiation of tumor subtypes–correlation with pathologic findings*. Radiology, 2009. 250(3): p. 793–802. 19244046 10.1148/radiol.2503080995

[32] Oliva, M.R., J.N. Glickman, K.H. Zou, et al., *Renal cell carcinoma: t1 and t2 signal intensity characteristics of papillary and clear cell types correlated with pathology*. AJR Am J Roentgenol, 2009. 192(6): p. 1524–1530. 19457814 10.2214/AJR.08.1727

[33] Couvidat, C., D. Eiss, V. Verkarre, et al., *Renal papillary carcinoma: CT and MRI features*. Diagn Interv Imaging, 2014. 95(11): p. 1055–1063. 25443332 10.1016/j.diii.2014.03.013

[34] Sasiwimonphan, K., N. Takahashi, B.C. Leibovich, et al., *Small (< 4 cm) Renal Mass: Differentiation of Angiomyolipoma without Visible Fat from Renal Cell Carcinoma Utilizing MR Imaging*. Radiology, 2016. 280(2): p. 653. 27429151 10.1148/radiol.2016164024

